# Developmental plasticity: Need to go beyond naïve thinking

**DOI:** 10.1093/emph/eox020

**Published:** 2018-02-05

**Authors:** Milind Watve

**Affiliations:** Department of Biology, Indian Institute of Science Education and Research, Pashan, Pune 411008, India

**Keywords:** developmental plasticity; fetal origins of adulthood disease; predictive adaptive response; factorial design

Early life constraints affecting phenotype in later life is a trend consistently observed across different species. Of particular relevance to evolutionary medicine are the effects of intra-uterine growth leading to diabetes, hypertension, cardiovascular disease and other age-related disorders. The broader question of conditions under which such developmental plasticity or predictive adaptive response (PAR) can evolve and further whether individual variability in plasticity will evolve have been theoretically addressed only recently [1–3]. Lea *et al.* [4], in this issue, expose some of the gaps and ambiguities in the concept, suggest experimental approaches and explore possible proximate mechanisms of plasticity. Being able to effectively bridge the proximate and ultimate would be the ultimate goal of understanding developmental plasticity. But currently there are many fundamental questions haunting the field.

One is that of clearly classifying early life effects and being able to resolve between them. A variety of attempts to define and classify are suggested including developmental constraints (DCs) versus PAR, internal PAR versus external PAR [2] and developmental versus activational plasticity [5]. Although there are attempts to segregate DC from PAR, the two are not mutually exclusive and can also mask each other’s effects making empirical testing difficult. Lea *et al.* [6] argued that since baboons born in a famine year are not better adapted to subsequent famine, the PAR hypothesis is not supported. However interpreting such observations is not simple [7]. If the adaptive component of the phenotype or the proximate mechanism is well-defined, a more rigorous experimental design [2, 7] is possible and desirable. A comparison of the fitness of individuals born with a constraint and developing the adaptive phenotype with individuals born with the constraint but without the phenotype would reflect on PAR reliably [2]. In the absence of this dimension, the match-mismatch experiment is inadequate to test PAR.

Further, since there are many alternative paths from early life input to a resultant phenotype and more than one path may be operational in a given example, I would suggest a flow chart representation (Fig. 1) rather than a dichotomous classification. For multiple pathways, a factorial design may be inadequate and a path analysis approach or a multiple predictions approach to test many alternative hypotheses [8] might prove better.

**Figure 1.**
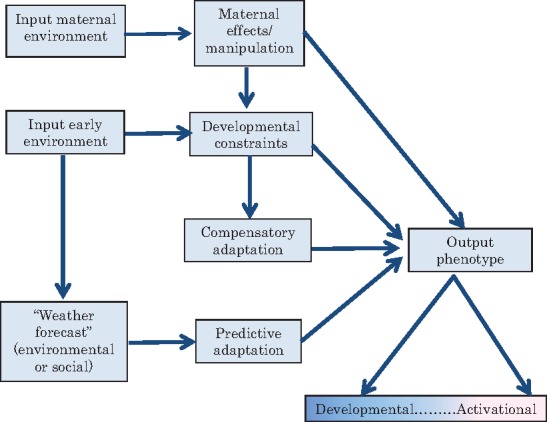
**Pathways of developmental plasticity:** Since none of the pathways are mutually exclusive and many pathways could be operational simultaneously, rather than any dichotomous classification, a path analysis approach or multiple predictions approach would work better for empirically testing the phenomenon. The developmental-activational plasticity is a continuum rather than a dichotomy and evolution along this continuum would depend upon the cost of reversing the phenotype versus the possible loss by mismatch

## THE MULTIDIMENSIONAL NATURE OF ADAPTATION

The other issue is about identifying what is the possible adaptation. There can be multiple challenges posed by an early life constraint and there can be multiple solutions to salvage the fitness loss. But the current thinking behind the factorial design is too simplistic. It is likely that PAR on facing a ‘famine’ in early life is not for facing another ‘famine’ later but for something else. For example, early life nutrition or maternal investment may affect male display in adult life [9] and for males with constrained display, it might be better to try an alternative reproductive strategy (ARS) such as sneak-mating. The physiological demands of an aggressive male and a sneaky male can be very different and there may be PAR addressing this. Insulin signalling is shown to play a role in courtship displays and reproductive behaviors over a wide range of taxa [10, 11] and therefore DCs may modulate insulin signalling differentially to adapt to a reproductive challenge. An aggressive male is more likely to take up fights and receive injuries more frequently than a weaker male who gives submissive displays and avoids a fight. Thus the immune system may be fine-tuned differently. Since the reproductive opportunities are different, parental investment and other life history strategies can also be substantially different and accordingly the hormonal make up. A change in one strategy thus needs fine tuning of many other systems and therefore the systems have evolved a complex signalling network [12].

Male ARS is just one example. There can be many more ways in which DC poses different nutritional, ecological, social or reproductive challenges and the physiology can be adaptively programmed accordingly. Therefore, our current perception of ‘match’ or ‘mismatch’ could be completely misguided. Early life ‘famine’ need not program the body for a future ‘famine’, but for a socially subordinate life and the social, nutritional, ecological, reproductive, physiological, immunological and other associated challenges. Unless we take into account many alternative adaptive strategies, a PAR hypothesis cannot be experimentally rejected by a match/mismatch factorial experiment. The inability to perceive alternative hypotheses might come from the fragmentation of biology as a discipline and inadequate dialogue among researchers with different directions of thinking.

## THE IMPORTANCE OF ‘JUST SO…’ STORIES

Evolutionary biology has undergone large amplitude oscillations in its approach. At some stage, it was laden with many unsupported ‘just so’ stories. Today the field has swung to the other extreme where we do not entertain any hypothesis unless it already has ‘convincing’evidence. This contributes largely to a deficiency of alternative hypotheses and under this deficiency we are forced into the trap of naïve thinking.

Lea *et al.* [1] also discuss the possible genes and molecules involved in developmental programming. This is certainly a welcome direction although at the moment insights into mechanisms of plasticity are few. We don’t need to wait for complete clarity on the PAR hypothesis in order to look for proximate mechanisms. In fact, having transcriptomic or any other omic correlates of a given putative PAR can suggest more alternative adaptive hypotheses. Any omics study always gives multiple hits, most of which remain uninterpreted. The underutilization of the omics data can at least partly be due to our failure to understand the multidimensional nature of adaptations. It is likely that the mechanisms of developmental plasticity are related to the network structure than to one or a few genes and molecules [12]. Pursuing both proximate and ultimate levels of investigation into developmental plasticity is likely to give us some useful clinical breakthroughs, which should be treated as the ultimate marker of success of evolutionary medicine.

**Conflict of interest:** None declared.
